# Author Correction: Taxonomic identification and temperature stress tolerance mechanisms of Aequorivita marisscotiae sp. nov

**DOI:** 10.1038/s42003-024-06257-8

**Published:** 2024-05-07

**Authors:** Wenqi Liu, Bailin Cong, Jing Lin, Shenghao Liu, Aifang Deng, Linlin Zhao

**Affiliations:** First Institute of Oceanography, Ministry of Natural Resources, Qingdao, 266061 China

**Keywords:** Marine microbiology, Bacterial evolution, Water microbiology, Bacterial transcription

Correction to: *Communications Biology* 10.1038/s42003-023-05559-7, published online 21 November 2023

In this article the funding from ‘The China-ASEAN Blue Partnership Construction Program, grant/award numbers: WJ1324013 and WJ1324015’ was omitted. The original article has been corrected.

