# Supplementary material for: Taxonomic identification and temperature stress tolerance mechanisms of Aequorivita marisscotiae sp. nov
Source: Commun Biol. 2023 Nov 21;6:1186. doi: 10.1038/s42003-023-05559-7 (PMC10663628; doi:10.1038/s42003-023-05559-7)
Supplement: Supplementary file 2 — Supplementary Information [file 42003_2023_5559_MOESM2_ESM.pdf]

Supplementary Information for

Taxonomic identification and mechanisms tolerance to temperature stress of the *Aequorivita marisscotiae* sp. nov.

Wenqi Liu[1,2], Bailin Cong[1,2]*, Jing Lin[1], Shenghao Liu[1], Aifang Deng[1], Linlin Zhao[1]

1. *First Institute of Oceanography, Ministry of Natural Resources, Qingdao, 266061, China.*

2 These authors contributed equally.

* Corresponding author: biolin@fio.org.cn.

This file includes four supplementary tables:

Supplementary Table 1 The result of average nucleotide identity analysis. Strains: 1, *Aequorivita marisscotiae* Ant34-E75; 2, *Aequorivita viscosa* 8-1b^{T}; 3, *Aequorivita lutea* q18^{T}; 4, *Aequorivita sinensis* S1-10^{T}; 5, *Aequorivita iocasae* KX20305^{T}; 6, *Aequorivita aquimaris* D-24^{T}; 7, *Aequorivita echinoideorum* CC-CZW007^{T}; 8, *Aequorivita vladivostokensis* KMM 3516^{T}; 9, *Aequorivita antarctica* SW49^{T}; 10, *Aequorivita lipolytica* Y10-2^{T}; 11, *Aequorivita soesokkakensis* RSSK-12^{T}.

Supplementary Table 2 Summary of RNA-seq data.

Supplementary Table 3 Summary of reads mapped to the reference genome.

Supplementary Table 4 Representative temperature stress-related differentially expressed genes. Ratio: upregulated genes/differential genes.

Supplementary Table 5 Representative temperature stress-related genes of the modules.

Supplementary Table 6 The primers sequences of the genes.

Supplementary Table 7 The relative mRNA levels of Quantitative real-time RT–PCR under different conditions.

Supplementary Table 1 The result of average nucleotide identity analysis. Strains: 1, *Aequorivita marisscotiae* Ant34-E75; 2, *Aequorivita viscosa* 8-1b^{T}; 3, *Aequorivita lutea* q18^{T}; 4, *Aequorivita sinensis* S1-10^{T}; 5, *Aequorivita iocasae* KX20305^{T}; 6, *Aequorivita aquimaris* D-24^{T}; 7, *Aequorivita echinoideorum* CC-CZW007^{T}; 8, *Aequorivita vladivostokensis* KMM 3516^{T}; 9, *Aequorivita antarctica* SW49^{T}; 10, *Aequorivita lipolytica* Y10-2^{T}; 11, *Aequorivita soesokkakensis* RSSK-12^{T}.

ANI (%)	1	2	3	4	5	6	7	8	9	10	11
1	*	75.05	75.85	75.80	77.99	78.65	73.96	78.30	78.13	78.09	78.17
2		*	73.04	75.69	74.63	74.46	72.88	74.29	75.54	75.02	75.27
3			*	73.29	78.23	78.59	74.21	78.77	77.03	76.33	78.31
4				*	75.37	75.19	72.90	74.75	76.04	75.82	75.85
5					*	83.83	75.44	81.74	79.80	79.87	81.09
6						*	75.08	89.54	79.86	79.96	81.34
7							*	75.43	74.96	74.90	75.65
8								*	79.20	79.26	80.47
9									*	80.70	81.36
10										*	80.33
11											*

Supplementary Table 2 Summary of RNA-seq data.

Sample	Raw Reads	Clean Reads	Clean Base (G)	Q20 (%)	Q30 (%)	G+C Content (mol%)
27_1	19,857,462	19,586,188	2.81	98.82	95.34	45.25
27_2	18,636,426	18,394,324	2.62	98.96	95.78	46.12
27_3	19,030,204	18,760,502	2.60	99.05	96.06	46.44
37-2_1	19,252,708	18,986,276	2.67	98.97	95.82	46.15
37-2_2	20,354,208	20,093,650	2.80	99.01	95.97	46.20
37-2_3	24,557,324	24,232,476	3.41	99.03	96.02	46.21
37-6_1	18,211,812	17,931,468	2.51	98.94	95.72	45.17
37-6_2	17,260,268	17,002,266	2.39	98.82	95.37	44.06
37-6_3	16,731,564	16,464,286	2.31	98.97	95.83	44.84
5-2_1	15,081,602	14,854,326	2.14	98.82	95.31	45.17
5-2_2	16,878,014	16,648,740	2.38	98.90	95.61	45.45
5-2_3	15,362,702	15,149,050	2.18	98.90	95.61	45.77
5-6_1	18,236,300	17,984,832	2.59	98.87	95.49	46.42
5-6_2	17,634,972	17,383,664	2.49	98.93	95.70	47.10
5-6_3	17,332,656	17,096,290	2.42	98.94	95.79	46.71

Supplementary Table 3 Summary of reads mapped to the reference genome.

Sample	Total reads	Total mapped	Mutiple mapped	Uniquely mapped	Reads map to '+'	Reads map to '-'
27_1	14,818,030	14,700,800（99.21%）	4,125,919（27.84%）	10,574,881（71.36%）	5,286,911（35.68%）	5,287,970（35.69%）
27_2	13,589,476	13,476,230（99.17%）	4,808,677（35.39%）	8,667,553（63.78%）	4,333,152（31.89%）	4,334,401（31.90%）
27_3	12,059,066	11,925,509（98.89%）	3,430,951（28.45%）	8,494,558（70.44%）	4,247,385（35.22%）	4,247,173（35.22%）
37-2_1	13,045,086	12,924,656（99.08%）	4,386,021（33.62%）	8,538,635（65.45%）	4,269,231（32.73%）	4,269,404（32.73%）
37-2_2	13,717,582	13,582,212（99.01%）	4,644,802（33.86%）	8,937,410（65.15%）	4,468,621（32.58%）	4,468,789（32.58%）
37-2_3	17,989,196	17,836,516（99.15%）	5,290,427（29.41%）	12,546,089（69.74%）	6,273,053（34.87%）	6,273,036（34.87%）
37-6_1	13,359,120	13,250,877（99.19%）	2,911,437（21.79%）	10,339,440（77.40%）	5,169,987（38.70%）	5,169,453（38.70%）
37-6_2	13,257,080	13,155,315（99.23%）	2,291,269（17.28%）	10,864,046（81.95%）	5,431,608（40.97%）	5,432,438（40.98%）
37-6_3	12,577,280	12,462,444（99.09%）	2,452,334（19.50%）	10,010,110（79.59%）	5,005,202（39.80%）	5,004,908（39.79%）
5-2_1	12,776,910	12,683,431（99.27%）	3,045,983（23.84%）	9,637,448（75.43%）	4,817,461（37.70%）	4,819,987（37.72%）
5-2_2	12,251,682	12,138,684（99.08%）	3,845,876（31.39%）	8,292,808（67.69%）	4,146,296（33.84%）	4,146,512（33.84%）
5-2_3	12,196,646	12,118,199（99.36%）	4,190,722（34.36%）	7,927,477（65.00%）	3,963,130（32.49%）	3,964,347（32.50%）
5-6_1	14,076,438	13,973,412（99.27%）	5,760,283（40.92%）	8,213,129（58.35%）	4,105,712（29.17%）	4,107,417（29.18%）
5-6_2	13,180,484	13,074,212（99.19%）	5,920,890（44.92%）	7,153,322（54.27%）	3,575,927（27.13%）	3,577,395（27.14%）
5-6_3	12,092,718	11,986,149（99.12%）	5,577,065（46.12%）	6,409,084（53.00%）	3,204,148（26.50%）	3,204,936（26.50%）

Supplementary Table 4 Representative temperature stress-related differentially expressed genes. Ratio: upregulated genes/differential genes.

Function	Pathway	27 vs 5-2		27 vs 5-6		27 vs 37-2		27 vs 37-6	
		Log_2 Fold Change	Ratio	Log_2 Fold Change	Ratio	Log_2 Fold Change	Ratio	Log_2 Fold Change	Ratio
Glycan biosynthesis and metabolism	Other glycan degradation	1.40	1/1	1.79	1/1	~	~	~	~
	Peptidoglycan biosynthesis	1.24 ~ 1.32	2/2	1.25 ~ 1.70	6/6	~	~	-2.00 ~ -1.38	0/4
Lipid metabolism	Fatty acid biosynthesis	1.17	1/1	~	~	~	~	-1.68	0/1
	Fatty acid degradation	~	~	1.87	1/1	~	~	1.04	1/1
Environmental Information Processing	ABC transporters	-1.79 ~ 1.27	2/6	-1.95 ~ 1.67	3/7	-1.35	0/1	-1.60 ~ -1.41	0/4
	Two-component system	-2.96 ~ -1.98	0/5	-4.25 ~ -1.41	0/6	-1.51 ~ 1.72	2/3	-2.15 ~ 1.81	1/3
Genetic Information Processing	RNA degradation	-1.46 ~ -4.04	0/2	-3.05 ~ 1.26	1/3	1.24	1/1	-2.83 ~ 1.96	1/3
	DNA replication	~	~	1.33	1/1	1.60	1/1	-2.06 ~ 1.49	1/3
	Homologous recombination	1.47	1/1	1.05	1/1	~	~	-1.56 ~ -1.27	0/2
	Aminoacyl-tRNA biosynthesis	-1.19	0/1	-1.63 ~ -1.07	0/3	1.05	1/1	-1.93 ~ 1.35	2/3
	Ribosome	-1.92 ~ -1.01	0/24	-2.89 ~ -1.07	0/48	-2.35 ~ 1.74	7/10	-2.16 ~ 2.33	27/30
Cellular Processes	Bacterial chemotaxis	1.85	1/1	2.83	1/1	1.49 ~ 2.53	2/2	1.80	1/1
	Quorum sensing	-1.18 ~ -1.02	0/3	-1.78 ~ 1.49	1/4	~	~	-1.29 ~ 1.53	3/4

34 Supplementary Table 4 (Continued)

Function	Pathway	27 vs 5-2		27 vs 5-6		27 vs 37-2		27 vs 37-6	
		Log_2 Fold Change	Ratio	Log_2 Fold Change	Ratio	Log_2 Fold Change	Ratio	Log_2 Fold Change	Ratio
Amino acid metabolism	Alanine, aspartate and glutamate metabolism	-2.37 ~ -2.01	2/3	-3.50 ~ 3.02	2/3	1.32	1/1	1.27	1/1
	Arginine and proline metabolism	~	~	-1.07	0/1	1.77 ~ 2.99	3/3	1.88 ~ 2.51	2/2
	Arginine biosynthesis	~	~	~	~	-1.74	0/1	-1.62	0/1
	Cysteine and methionine metabolism	-1.37	0/1	-3.69 ~ -1.04	0/7	1.81	1/1	1.27 ~ 2.48	3/3
	Glycine, serine and threonine metabolism	1.36 - 3.23	5/5	1.28 ~ 4.08	3/3	~	~	-1.98 ~ 3.64	2/3
	Histidine metabolism	-2.43 ~ 1.33	2/7	-3.44 ~ 1.56	2/8	1.61	1/1	1.12	1/1
	Lysine biosynthesis	~	~	1.11 ~ 1.38	2/2	~	~	-2.00	0/1
	Phenylalanine metabolism	1.45	1/1	1.13 ~ 1.33	3/3	~	~	~	~
	Phenylalanine, tyrosine and tryptophan biosynthesis	~	~	~	~	1.38	1/1	-2.22 ~ 3.34	7/9
	Valine, leucine and isoleucine biosynthesis	-1.87 ~ -1.53	0/2	-4.18 ~ -3.57	0/2	~	~	~	~
	Tryptophan metabolism	~	~	~	~	~	~	-1.75	0/1
	Valine, leucine and isoleucine degradation	1.34	1/1	-1.30 ~ 1.74	3/4	-1.20	0/1	-1.54 ~ 1.32	1/2

Supplementary Table 4 (Continued)

Function	Pathway	27 vs 5-2		27 vs 5-6		27 vs 37-2		27 vs 37-6	
		Log2 Fold Change	Ratio	Log2 Fold Change	Ratio	Log2 Fold Change	Ratio	Log2 Fold Change	Ratio
Other metabolism	Streptomycin biosynthesis	1.15	1/1	1.31	1/1	~	~	~	~
	Amino sugar and nucleotide sugar metabolism	~		1.22	1/1	~	~	~	~
	Citrate cycle (TCA cycle)	~		1.42	1/1	~	~	~	~
	Glycolysis / Gluconeogenesis	-1.20 ~ 1.16	1/2	-1.47 ~ 1.10	1/2	1.09 ~ 1.15	2/2	-1.93 ~ 1.33	2/4
	Nitrogen metabolism	1.27	1/1	1.39 ~ 2.50	3/3	1.04 ~ 1.54	2/2	1.27	1/1
	Oxidative phosphorylation	-1.54 ~ 1.39	1/4	-1.66 ~ 1.19	2/6	1.20 ~ 1.23	2/2	1.04 ~ 2.29	7/7
	Folate biosynthesis	1.06	1/1	-1.05 ~1.33	1/2	~	~	~	~
	Nicotinate and nicotinamide metabolism	1.17 - 1.30	3/3	1.39 ~ 2.43	4/4	-1.30 ~ -1.29	0/2	-1.49 ~ -1.50	0/2
	Porphyrin and chlorophyll metabolism	-1.38 ~ 1.24	2/3	-1.69 ~ 1.81	1/3	~	~	1.46	1/1
	Ubiquinone and other terpenoid-quinone biosynthesis	~	~	-1.01 ~ 1.35	3/4	-1.28	0/1	~	~
	D-Glutamine and D-glutamate metabolism	~	~	1.11 ~ 1.70	2/2	~	~	-1.90	0/1
	Selenocompound metabolism	~	~	1.38	1/1	~	~	-1.69	0/1
	Purine metabolism	-3.66	0/1	-4.04 ~ 1.80	3/11	-1.40 ~ 1.21	1/3	-2.41 ~ 1.78	1/7
	Pyrimidine metabolism	1.53	1/1	1.50 ~ 1.62	2/2	~	~	-1.53 ~ 1.16	2/3

Supplementary Table 5 Representative temperature stress-related genes of the modules.

Module	Gene id	weight	Gene symbol	Functional annotation	log_2 Fold Change			
					27 vs 5-2	27 vs 5-6	27 vs 37-2	27 vs 37-6
	E75_GM003126	104.55	*rpl*I	50S ribosomal protein L9	-1.20	-1.77	~	1.20
	E75_GM000233	96.85	*rpm*C	50S ribosomal protein L29	-1.64	-1.76	~	~
	E75_GM000234	97.47	*rpl*P	50S ribosomal protein L16	-1.40	-1.77	~	1.42
	E75_GM000242	98.01	*rps*J	30S ribosomal protein S10	-1.01	-1.22	~	1.12
	E75_GM000244	97.33	*rps*G	30S ribosomal protein S7	~	-1.39	~	1.61
	E75_GM001213	95.21	*rpl*Y/Ctc	50S ribosomal protein L25/general stress protein Ctc	-1.28	-1.29	1.18	2.29
	E75_GM001098	98.93	*ecs*B	Bacterial ABC transporter protein	-1.74	-1.80	~	1.68
	E75_GM003173	98.33	*ykp*A	ABC transporter ATP-binding protein	-1.54	-1.44	~	1.07
	E75_GM000190	99.33	ABCF3	ATPase component of ABC transporters with duplicated ATPase domain	-1.61	-1.60	1.55	1.63
Blue	E75_GM001834	108.53	*tuf*	Translation elongation factor 1A (EF-1A/EF-Tu)	-1.23	-1.68	~	1.53
	E75_GM001265	99.78	*tsf*	Translation elongation factor Ts (EF-Ts)	-2.18	-1.98	~	2.46
	E75_GM000243	112.22	*fus*A	Elongation factor G	-1.37	-1.77	~	1.36
	E75_GM000556	101.64	*lys*S	Lysyl-tRNA synthetase	-1.19	-1.63	~	~
	E75_GM001154	99.57	*hem*A	Glutamyl-tRNA reductase	-1.38	-1.69	~	~
	E75_GM001369	105.64	*yhc*X	Carbon-nitrogen hydrolase	~	-1.18	~	~
	E75_GM000933	101.46	*pgk*	Phosphoglycerate kinase	-1.20	-1.47	~	~
	E75_GM001301	97.49	EC1.9.3.1	Cytochrome C oxidoreductase	-1.05	-1.51	~	1.15
	E75_GM002848	97.82	*ych*F	Redox-regulated ATPase YchF	-1.75	-2.14	~	~
	E75_GM001835	95.50	*sec*E	Preprotein translocase subunit SecE	-1.18	-1.78	~	1.03
	E75_GM000134	100.54	*ndp*A	Nucleoid-associated protein NdpA	-1.12	-1.48	~	1.06

37 Supplementary Table 5 (Continued)

Module	Gene id	weight	Gene symbol	Functional annotation	log_2 Fold Change			
					27 vs 5-2	27 vs 5-6	27 vs 37-2	27 vs 37-6
Brown	E75_GM000267	66.42	*mur*C	UDP-N-acetylmuramate--L-alanine ligase	~	1.70	~	~
	E75_GM000364	60.56	*paa*E	Phenylacetic acid degradation protein	~	1.13	~	~
	E75_GM000543	62.96	*exb*D	Biopolymer transporter ExbD	1.39	1.61	~	~
	E75_GM000544	61.87	*exb*B	Biopolymer transporter ExbB	~	1.40	~	-1.26
	E75_GM000642	57.99	*gfd*R	DNA-binding response regulator	2.17	2.33	~	~
	E75_GM001774	64.84	RBP	RNA-binding protein	1.47	1.74	~	~
	E75_GM002198	58.95	SCO1/SenC/PrrC	Uncharacterized protein SCO1/SenC/PrrC, involved in biogenesis of respiratory and photosynthetic systems	1.24	2.07	~	~
	E75_GM002295	60.02	*sus*D	SusD family protein	1.69	2.23	~	-1.74
	E75_GM002361	64.76	*com*M	Magnesium chelatase	1.34	1.39	~	~
	E75_GM002377	59.53	*gpl*9	T4-like virus tail tube protein	3.30	3.83	~	~
	E75_GM002880	59.96	*rpo*E	RNA polymerase subunit sigma-24	1.81	1.78	~	-2.51
	E75_GM000537	64.83	~	Peptidase C25	1.78	2.10	~	~
	E75_GM000609	57.39	~	T9SS C-terminal target domain-containing protein	~	1.69	~	-1.60
	E75_GM001907	57.07	~	T9SS C-terminal target domain-containing protein	1.34	~	~	~
	E75_GM001271	57.90	~	Por secretion system C-terminal sorting domain-containing protein	1.24	~	~	~
	E75_GM000872	65.56	~	Hypothetical protein	1.77	2.35	~	~
	E75_GM002528	57.68	~	Hypothetical protein	3.45	2.64	~	~
	E75_GM002833	68.20	~	Hypothetical protein	1.56	1.98	~	-1.54
	E75_GM003171	66.97	~	Hypothetical protein	1.82	1.73	~	~
	E75_GM000308	64.65	~	~	2.05	2.55	~	~

Supplementary Table 6 The sequences of the genes primer.

Gene symbol	Gene id	Primer sequences
*mur*A	E75_GM003110	F: GAAGGCTAGACACCCACTTTGAAGG<br>R: TTGGGAGCTTCTACGCCGTAAAATC
*mur*B	E75_GM001659	F: ACGCCCAAACATTTCAAGACTTTCG<br>R: TGCACACCCGCATCACCAAAG
*mur*C	E75_GM000267	F: TTTACACTCCCGCAATTCCGAAGG<br>R: ACCCGCAACCGCTAAACACAG
*mur*D	E75_GM000270	F: GCCTGGAAGGATTTCAAGGAGTGG<br>R: GTGGCGTTTACATTGGTGGCTTTAG
*mur*E	E75_GM000272	F: CACCACGCCCGATTCACTGAC<br>R: TGTATGCCGTGCGAACTAACTTCC
*mur*F	E75_GM000539	F: AGCGAGATTGCAGATCCAGATTACG<br>R: TTACCTTGAACCACACCGTCTAAGC
*mra*Y	E75_GM000271	F: GGCTATCGCTGCGGTAATACTGTC<br>R: TTTGCCCATCCAACCCAAGATCG
*mur*G	E75_GM000268	F: AGAACAGTCACGCTGGAATCACG<br>R: TCGCCATGCCTTCATAAGCTACAC
*pbp*C	E75_GM001011	F: TTCAAACTTGCCCACCGTTCTCC<br>R: TCATTGCCTTCCGCATCCACAG
*mtg*A	E75_GM000980	F: GCCGAAGCAGCCTCACAATTTTG<br>R: ATTGGCACTATAGCGTTGTGGACTG
*bcr*C	E75_GM001902	F: AGCGCCATGGCCTTTTATG<br>R: TGGACGCCGAGATAAATTCG

Supplementary Table 7 The Relative mRNA levels of Quantitative real-time RT−PCR under different conditions.

Relative mRNA levels	27	37-2	37-4	37-6	37-8	37-10
		0.768437591	0.381297222	0.220962748	0.161878134	0.534032704
murF	1	0.773782497	0.40332088	0.25348987	0.184283652	0.566441943
		0.687770909	0.343885455	0.204475515	0.143587294	0.524858342
		0.744584098	0.577146851	0.303063099	0.036397925	0.048611874
bcrC	1	0.707106781	0.594603558	0.363493129	0.046070913	0.051474439
		0.784584098	0.582366793	0.248273124	0.052921582	0.0476956
		0.902637998	0.614584098	0.154780839	0.251249261	0.41754396
pbpC	1	0.936272247	0.584388624	0.182377543	0.224533093	0.452189689
		0.873572896	0.630688704	0.025826572	0.21538654	0.407536166
		0.986232704	0.637824968	0.152127255	0.189408115	0.297301779
mtgA	1	0.965936329	0.602903914	0.160428237	0.188155843	0.267943366
		0.907519155	0.594603558	0.135841858	0.188155843	0.348685917

Relative mRNA levels	27	5-2	5-4	5-6	5-8	5-10
		1.636816973	1.148698355	0.850927709	0.176776695	0.294369923
murA	1	1.735077374	1.218410264	0.909561678	0.165511114	0.257920795
		1.585568273	1.113421618	0.949591099	0.188809073	0.331022228
		1.351910833	1.109569472	1.064370182	0.146604369	0.305660069
murC	1	1.328685814	1.147335865	0.921964598	0.137738139	0.279321785
		1.375541818	1.070180406	0.840896415	0.156041319	0.334481889
		1.303298677	1.239522749	1.131773463	0.323088208	0.535886731
murD	1	1.380317353	1.010451446	0.936272247	0.321970407	0.501735874
		1.28788163	1.003471749	1.07549439	0.324209889	0.57236208
		1.305859787	1.180992661	1.176906737	0.345079338	0.586417475
murE	1	1.333298677	1.144724161	1.105730653	0.349896466	0.541487523
		1.448942155	1.218410264	1.252664439	0.340328529	0.635075491
		1.510472586	1.658639092	1.433955248	0.637280314	0.582366793
mraY	1	1.558329159	1.729074463	1.366040257	0.664342907	0.510506063
		1.464085696	1.591072968	1.319507911	0.611320139	0.664342907
		1.328685814	1.193335743	1.038859103	0.288171587	0.524858342
murG	1	1.337927555	1.197478705	0.888842681	0.279321785	0.47963206
		1.319507911	1.189207115	1.064370182	0.297301779	0.574349177
