# Supplementary material for: Taxonomic identification and temperature stress tolerance mechanisms of Aequorivita marisscotiae sp. nov
Source: Commun Biol. 2023 Nov 21;6:1186. doi: 10.1038/s42003-023-05559-7 (PMC10663628; doi:10.1038/s42003-023-05559-7)
Supplement: Supplementary file 3 — Description of Additional Supplementary Files [file 42003_2023_5559_MOESM3_ESM.docx]

Description of Additional Supplementary Files

**File name:** Supplementary Data 1

**Description:**The Summary of all differentially expressed genes of the strain Ant34-E75 and the source data behind Figure 2 in the paper.
